# Supplementary material for: S100A10 Promotes Pancreatic Ductal Adenocarcinoma Cells Proliferation, Migration and Adhesion through JNK/LAMB3-LAMC2 Axis
Source: Cancers (Basel). 2022 Dec 29;15(1):202. doi: 10.3390/cancers15010202 (PMC9818352; doi:10.3390/cancers15010202)
Supplement: Supplementary file 1 [file cancers-15-00202-s001.zip › Figure S1-7(Proofread).pdf]

Figure S1

S100	A1		A2		A3		A4		A5		A6		A7	
Analysis Type by Cancer	Cancer vs. Normal		Cancer vs. Normal		Cancer vs. Normal		Cancer vs. Normal		Cancer vs. Normal		Cancer vs. Normal		Cancer vs. Normal	
Bladder cancer			2								1			
Brain and CNS Cancer		7					2				2			
Breast Cancer		2						1				3	2	
Cervical Cancer														
Colorectal Cancer			16		3								1	
Esophageal Cancer				2		1								
Gastric Cancer			1				1							
Head and Neck Cancer	1	3	2		1			1		1		1	4	
Kidney Cancer		1		6	1						2			
Leukemia				1			3	3			1	4	2	
Liver Cancer											3			
Lung Cancer		1	7			8		12				1		
Lymphoma							4	1			5			
Melanoma	1			1										
Myeloma														
Other Cancer			1				4	1			1			
Ovarian Cancer	2		3								1			
Pancreatic Cancer			1				3				2			
Prostate Cancer								1				2		
Sarcoma		5			1		1	2			1	1		
Significant Unique Analyses	4	19	33	10	6	9	17	22		1	18	12	9	
Total Unique Analyses	349		335		342		309		300		243		320	

S100	A7A		A7B		A8		A9		A10		A11		A12	
Analysis Type by Cancer	Cancer vs. Normal		Cancer vs. Normal		Cancer vs. Normal		Cancer vs. Normal		Cancer vs. Normal		Cancer vs. Normal		Cancer vs. Normal	
Bladder cancer							1				3			
Brain and CNS Cancer					1				7		5			
Breast Cancer					2				1	1	2	1	1	
Cervical Cancer								1						1
Colorectal Cancer	1				4		4			2	15		1	
Esophageal Cancer						1		2		4		3		3
Gastric Cancer		1							3		1			
Head and Neck Cancer	3					1		2	2	1	1			
Kidney Cancer							3		4		6			
Leukemia					3	6	3	9	2	2	1	10		12
Liver Cancer						3			3		2			
Lung Cancer						8		3		6				2
Lymphoma									3	2	6			
Melanoma														
Myeloma						1		1						1
Other Cancer	2					1	1	1	2		6		2	1
Ovarian Cancer										1	1			
Pancreatic Cancer									4		4			
Prostate Cancer														
Sarcoma						8		2	1	2	1	1		
Significant Unique Analyses	6	1			10	29	12	21	29	21	54	15	4	20
Total Unique Analyses	153		22		344		326		352		314		344	

S100	A13		A14		A16		B		G		P		Z	
Analysis Type by Cancer	Cancer vs. Normal		Cancer vs. Normal		Cancer vs. Normal		Cancer vs. Normal		Cancer vs. Normal		Cancer vs. Normal		Cancer vs. Normal	
Bladder cancer			2					1			1			
Brain and CNS Cancer		2			1									
Breast Cancer	1		6		1			7			13			
Cervical Cancer				2							1			
Colorectal Cancer				7		2		2			11	1	1	
Esophageal Cancer		2		3		2					1	2		
Gastric Cancer												4		
Head and Neck Cancer				3							1	1		
Kidney Cancer				1		1								
Leukemia	5				2							9		
Liver Cancer			1								1			
Lung Cancer		1									3			
Lymphoma	8				1							3		
Melanoma				1			2					1		
Myeloma														
Other Cancer			1	1				1						
Ovarian Cancer	1		2								1			
Pancreatic Cancer	2		1		2						5			
Prostate Cancer		1		1		1		1						
Sarcoma		1		4				7				4		
Significant Unique Analyses	17	7	13	22	7	6	2	18			38	25	1	
Total Unique Analyses	343		248		171		362		296		344		147	

1 5 10 10 5 1

%

Box color is determined by the best gene rank percentile for the analyses within the box.
The numbers listed in each box represent the number of datasets that meet the filter criteria.

Figure S2

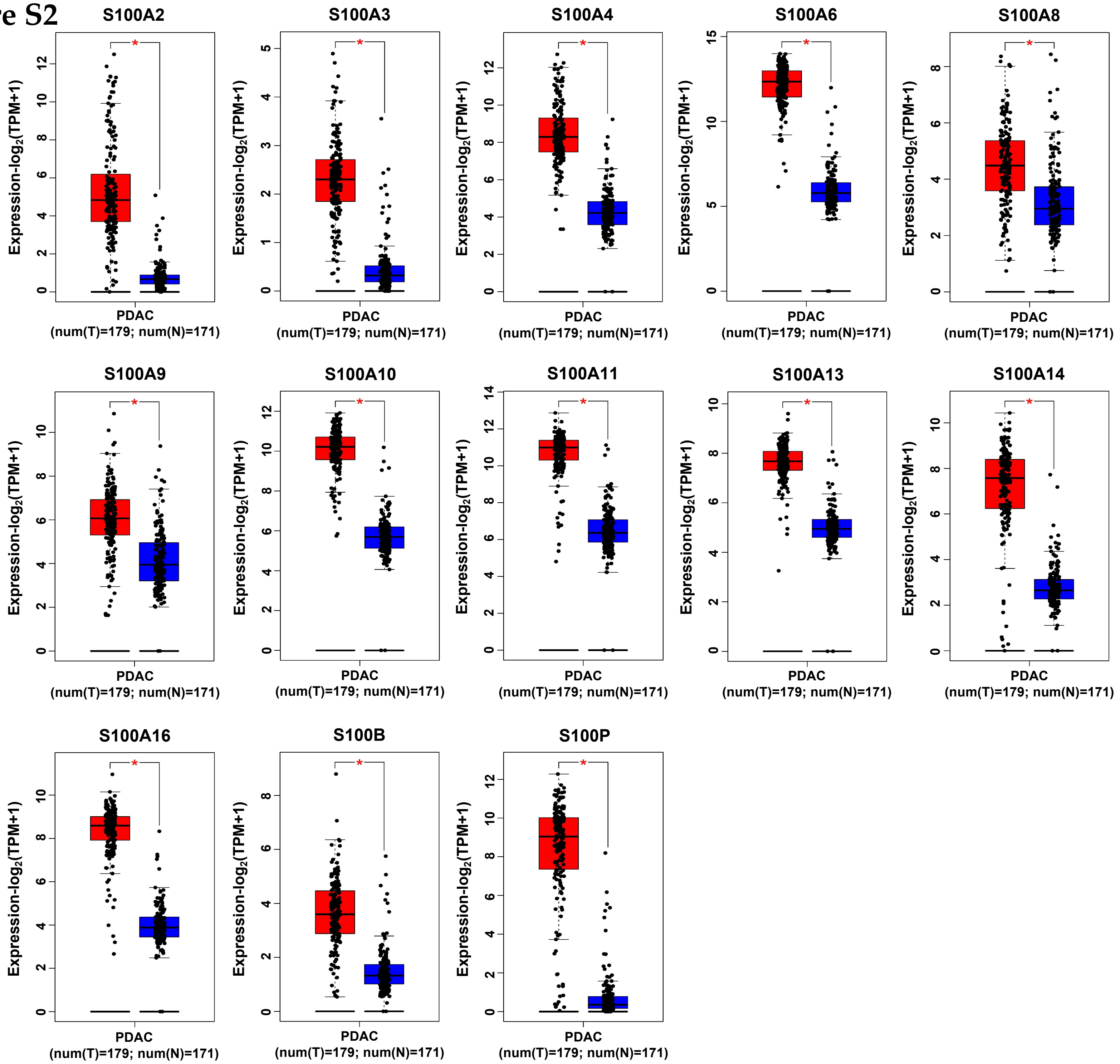

S100A2
S100A3
S100A4
S100A6
S100A8
S100A9
S100A10
S100A11
S100A13
S100A14
S100A16
S100B
S100P
Expression-log$_2$(TPM+1)
PDAC
(num(T)=179; num(N)=171)

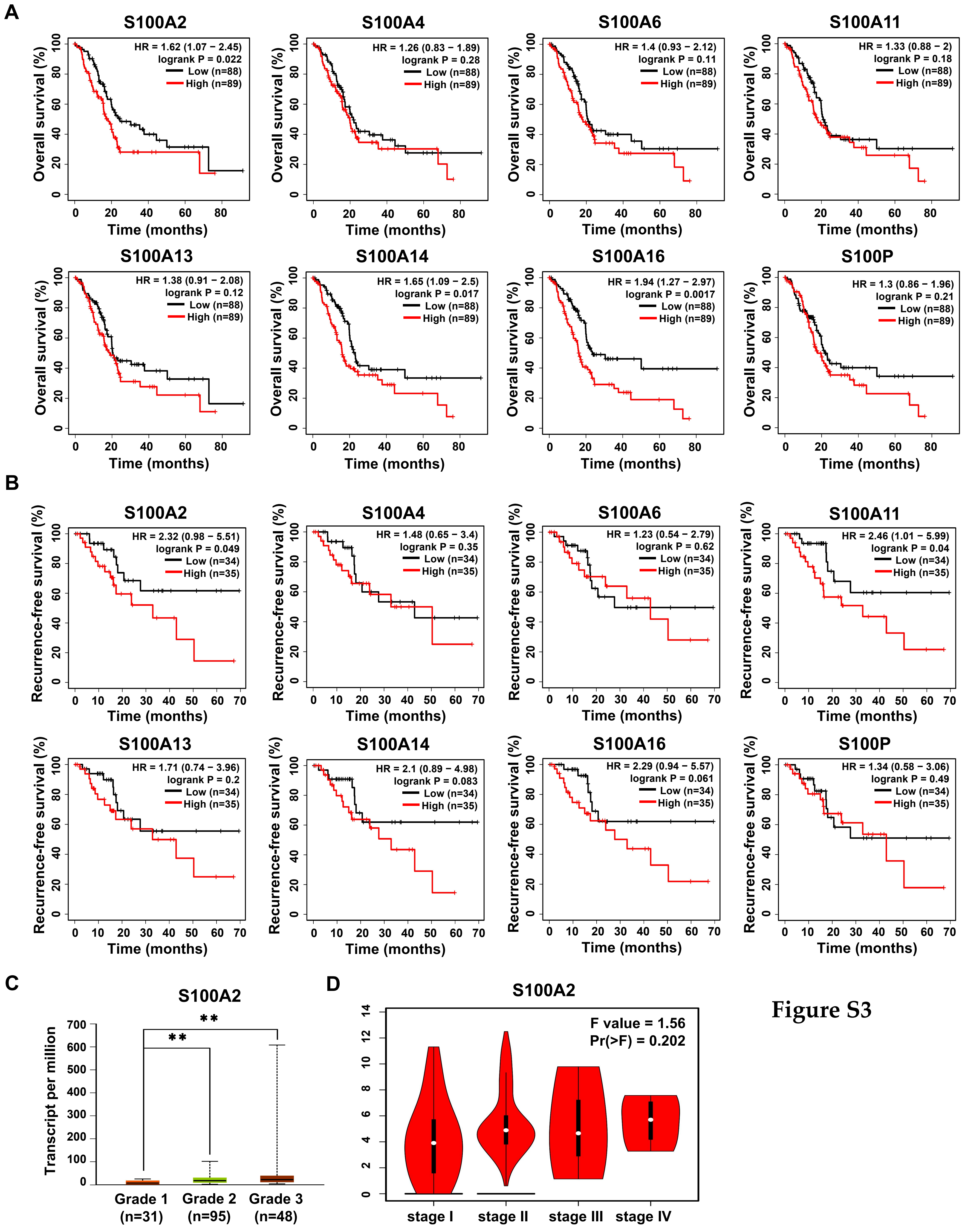
A
S100A2
HR = 1.62 (1.07 − 2.45)
logrank P = 0.022
Low (n=88)
High (n=89)
S100A4
HR = 1.26 (0.83 − 1.89)
logrank P = 0.28
S100A6
HR = 1.4 (0.93 − 2.12)
logrank P = 0.11
S100A11
HR = 1.33 (0.88 − 2)
logrank P = 0.18
S100A13
HR = 1.38 (0.91 − 2.08)
logrank P = 0.12
S100A14
HR = 1.65 (1.09 − 2.5)
logrank P = 0.017
S100A16
HR = 1.94 (1.27 − 2.97)
logrank P = 0.0017
S100P
HR = 1.3 (0.86 − 1.96)
logrank P = 0.21
Overall survival (%)
Time (months)
B
S100A2
HR = 2.32 (0.98 − 5.51)
logrank P = 0.049
Low (n=34)
High (n=35)
S100A4
HR = 1.48 (0.65 − 3.4)
logrank P = 0.35
S100A6
HR = 1.23 (0.54 − 2.79)
logrank P = 0.62
S100A11
HR = 2.46 (1.01 − 5.99)
logrank P = 0.04
S100A13
HR = 1.71 (0.74 − 3.96)
logrank P = 0.2
S100A14
HR = 2.1 (0.89 − 4.98)
logrank P = 0.083
S100A16
HR = 2.29 (0.94 − 5.57)
logrank P = 0.061
S100P
HR = 1.34 (0.58 − 3.06)
logrank P = 0.49
Recurrence-free survival (%)
Time (months)
C
S100A2
Transcript per million
**
Grade 1 (n=31)
Grade 2 (n=95)
Grade 3 (n=48)
D
S100A2
F value = 1.56
Pr(>F) = 0.202
stage I
stage II
stage III
stage IV

Figure S3

Figure S4

A

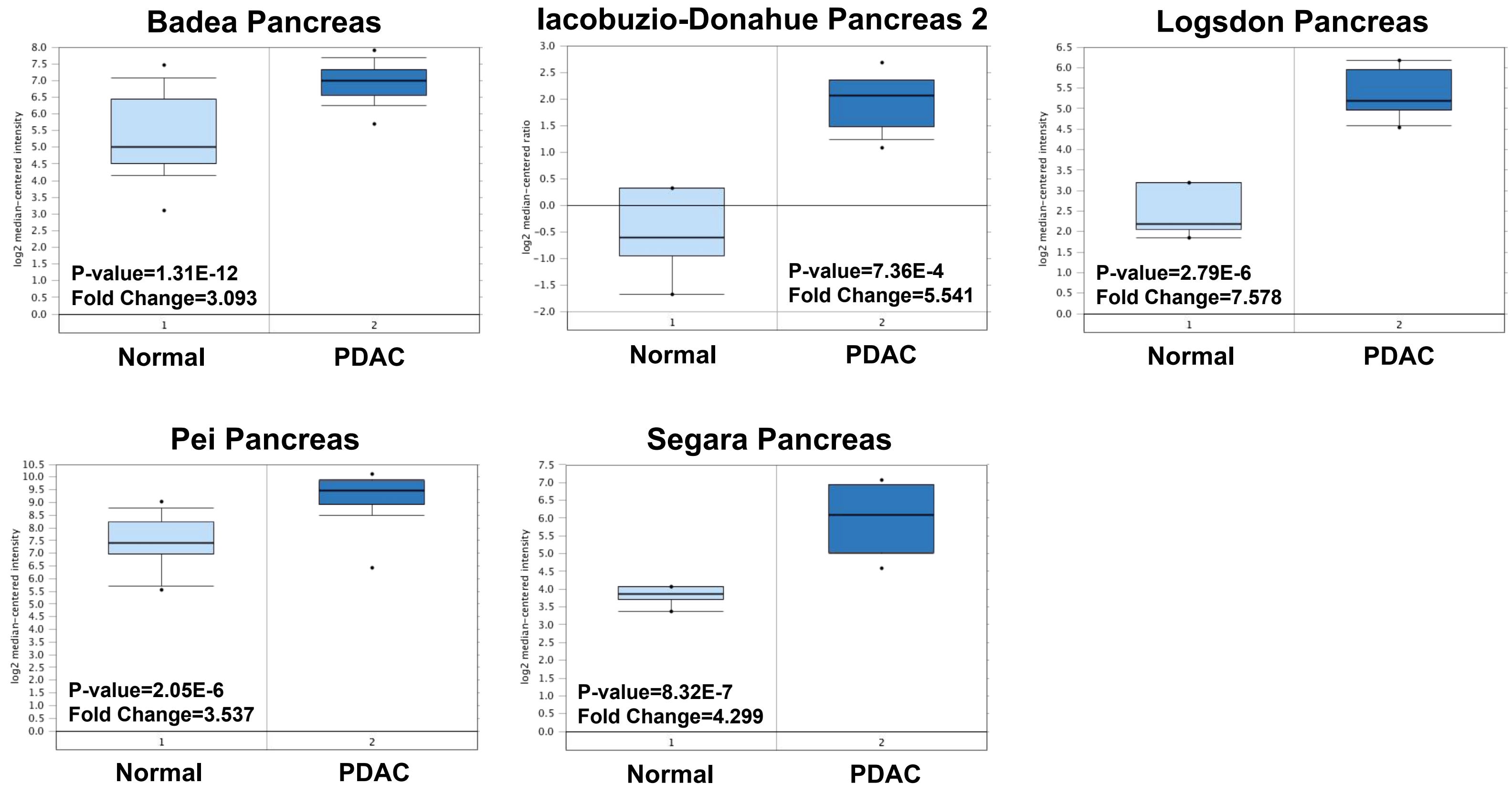

B

Comparision of S100A10 Across 5 Analyses

over-expression

Median Rank	p-Value	Gene	1	2	3	4	5
400.0	1.31E-11	S100A10					

Legend

1. Pancreatic Ductal Adenocarcinoma vs. Normal
   Badea Pancreas,Hepatogastroenterology, 2008
2. Pancreatic Adenocarcinoma vs. Normal
   Iacobuzio-Donahue Pancreas 2, Am J Pathol, 2003
3. Pancreatic Adenocarcinoma vs. Normal
   Logsdon Pancreas, Cancer Res, 2003
4. Pancreatic Carcinoma vs. Normal
   Pei Pancreas, Cancer Cell, 2009
5. Pancreatic Carcinoma vs. Normal
   Segara Pancreas, Clin Cancer Res, 2005

1 5 10 25 25 10 5 1 Not measured

← % →

The rank for a gene is the median rank for that gene across each of the analyses.
The p-Value for a gene is its p-Value for the median -rank analysis.

C

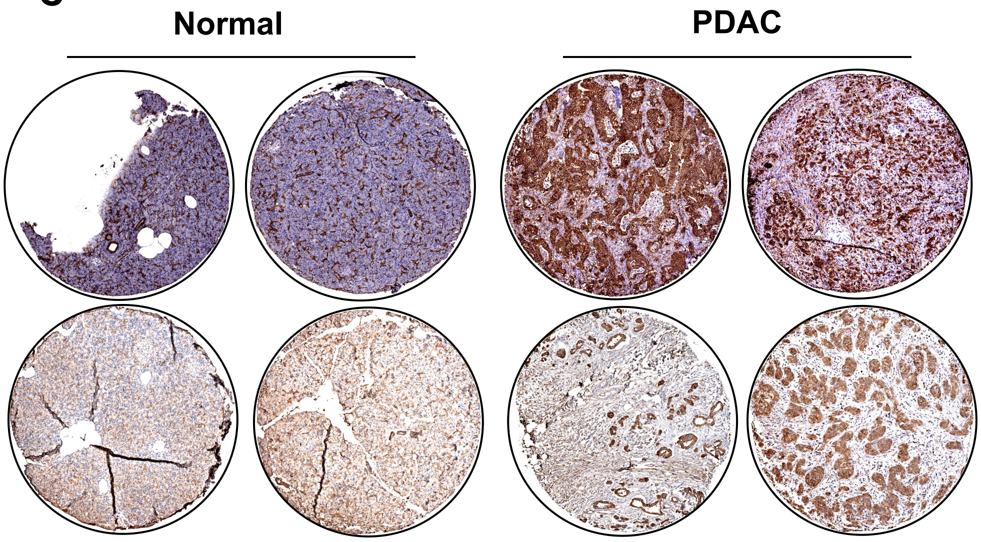

D

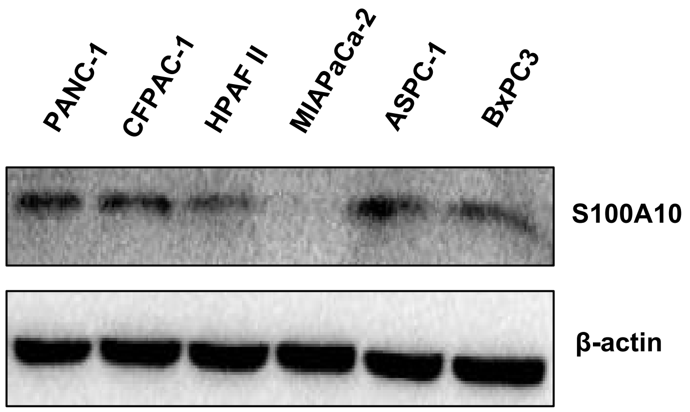

Figure S5

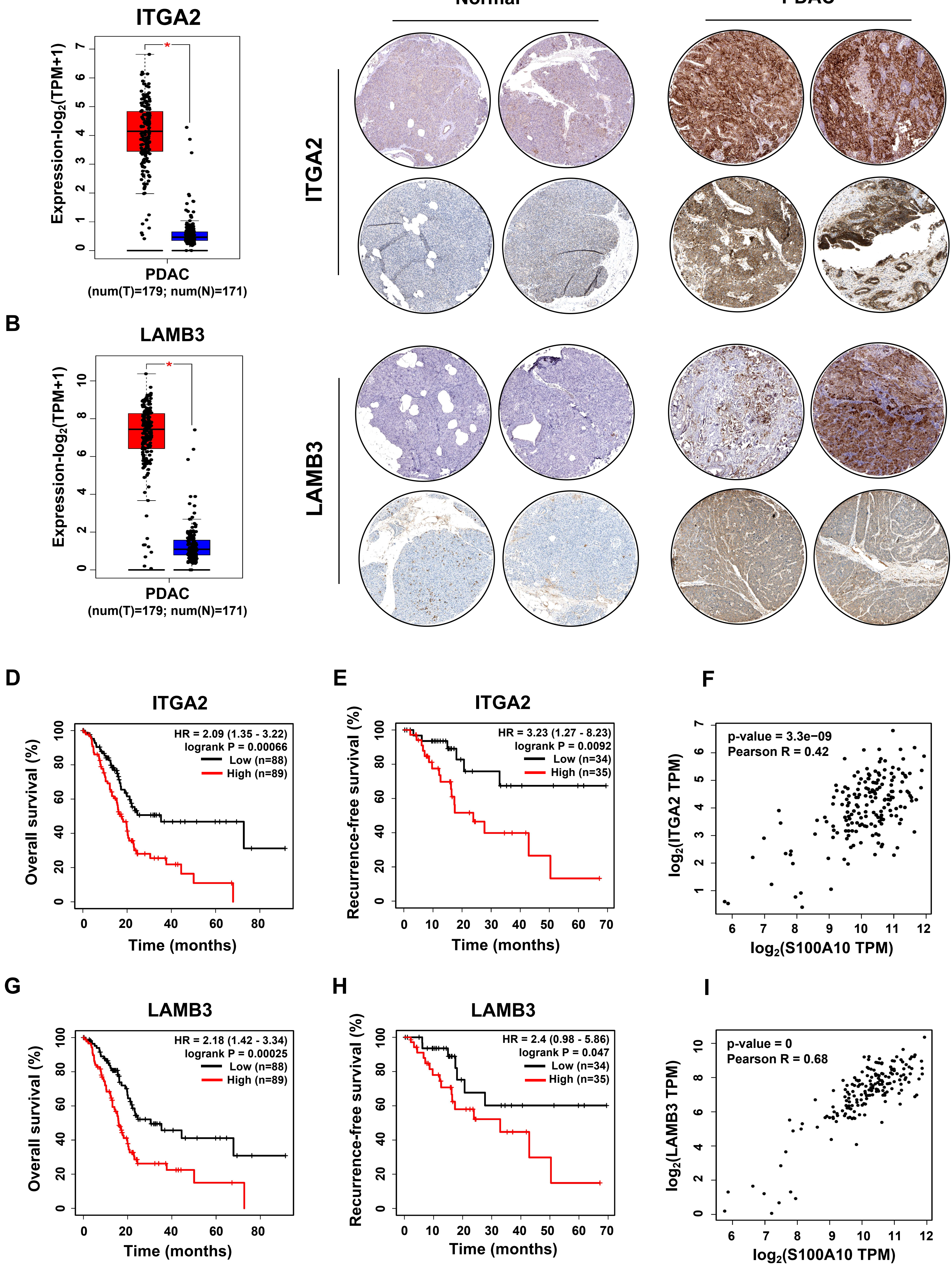
A
ITGA2
Expression-log2(TPM+1)
0 1 2 3 4 5 6 7
*
PDAC
(num(T)=179; num(N)=171)
B
LAMB3
Expression-log2(TPM+1)
0 2 4 6 8 10
*
PDAC
(num(T)=179; num(N)=171)
C
Normal
PDAC
ITGA2
LAMB3
D
ITGA2
HR = 2.09 (1.35 - 3.22)
logrank P = 0.00066
Low (n=88)
High (n=89)
Overall survival (%)
0 20 40 60 80 100
Time (months)
0 20 40 60 80
E
ITGA2
HR = 3.23 (1.27 - 8.23)
logrank P = 0.0092
Low (n=34)
High (n=35)
Recurrence-free survival (%)
0 20 40 60 80 100
Time (months)
0 10 20 30 40 50 60 70
F
p-value = 3.3e−09
Pearson R = 0.42
log2(ITGA2 TPM)
1 2 3 4 5 6 7
log2(S100A10 TPM)
6 7 8 9 10 11 12
G
LAMB3
HR = 2.18 (1.42 - 3.34)
logrank P = 0.00025
Low (n=88)
High (n=89)
Overall survival (%)
0 20 40 60 80 100
Time (months)
0 20 40 60 80
H
LAMB3
HR = 2.4 (0.98 - 5.86)
logrank P = 0.047
Low (n=34)
High (n=35)
Recurrence-free survival (%)
0 20 40 60 80 100
Time (months)
0 10 20 30 40 50 60 70
I
p-value = 0
Pearson R = 0.68
log2(LAMB3 TPM)
0 2 4 6 8 10
log2(S100A10 TPM)
6 7 8 9 10 11 12

Figure S6

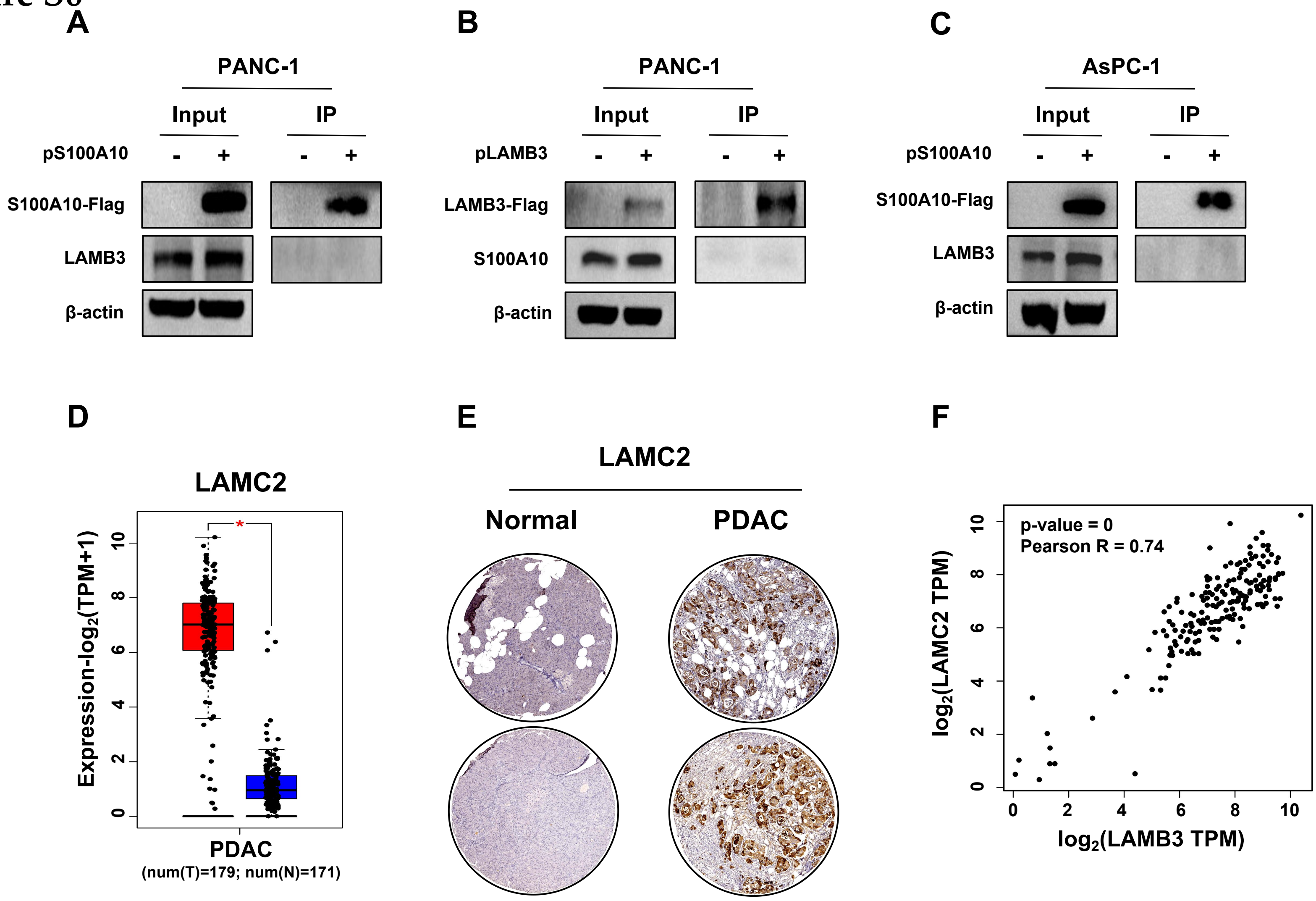
A
PANC-1
Input
IP
pS100A10
- + - +
S100A10-Flag
LAMB3
β-actin
B
PANC-1
Input
IP
pLAMB3
- + - +
LAMB3-Flag
S100A10
β-actin
C
AsPC-1
Input
IP
pS100A10
- + - +
S100A10-Flag
LAMB3
β-actin
D
LAMC2
Expression-log2(TPM+1)
*
PDAC
(num(T)=179; num(N)=171)
E
LAMC2
Normal
PDAC
F
p-value = 0
Pearson R = 0.74
log2(LAMC2 TPM)
log2(LAMB3 TPM)

Figure 2B

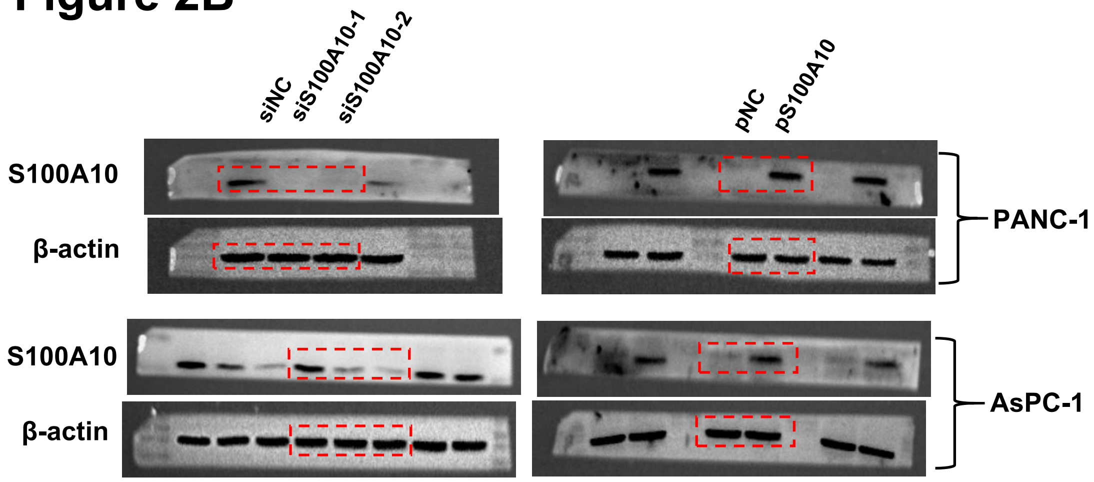

Figure 3B

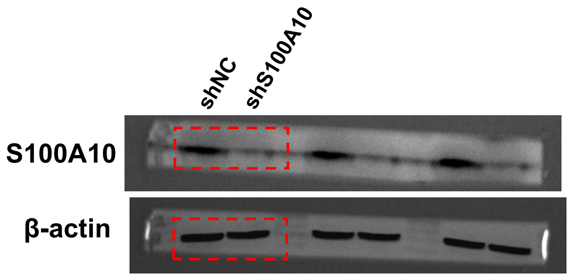

Figure 5A

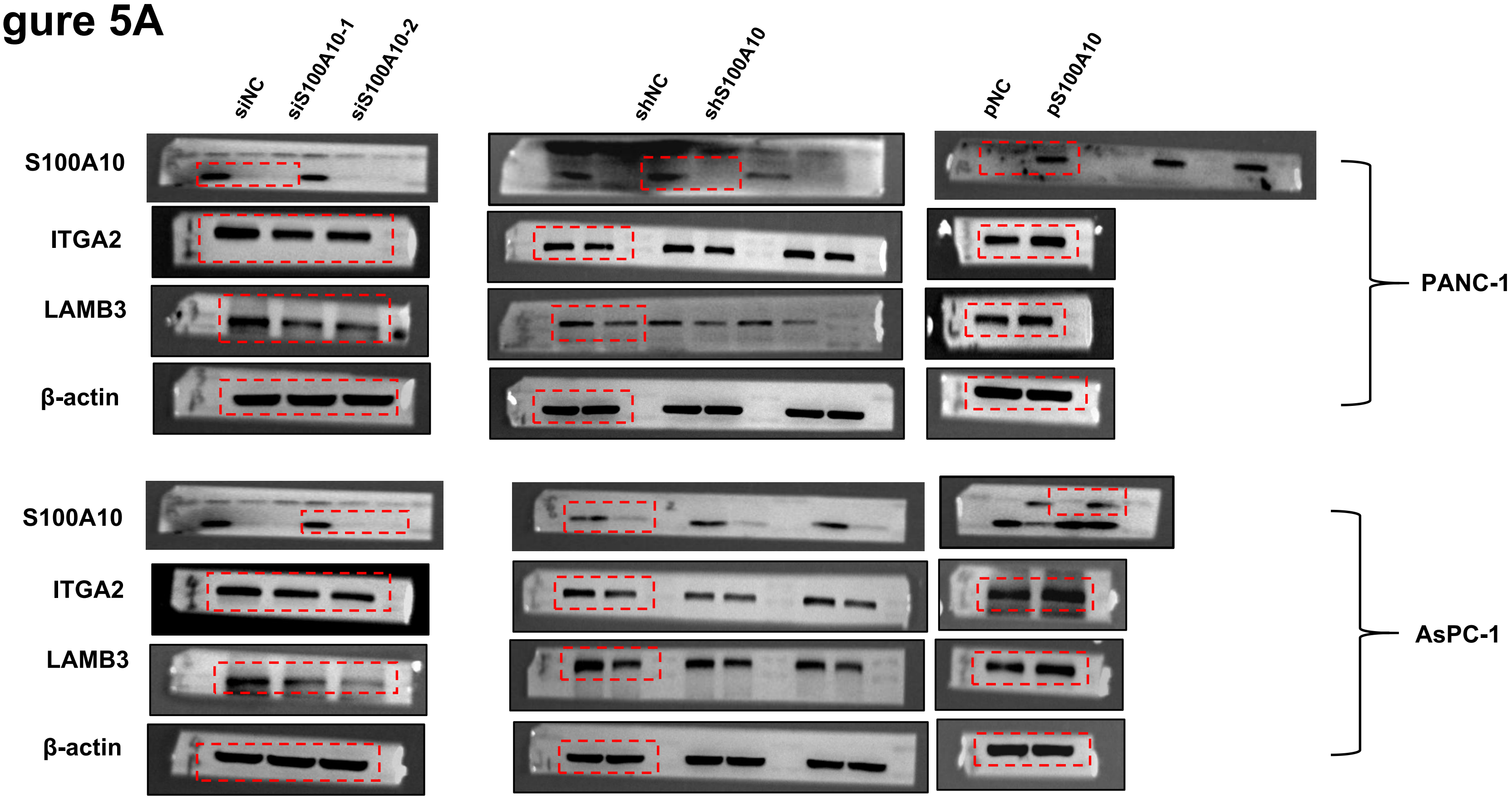

Figure 6B

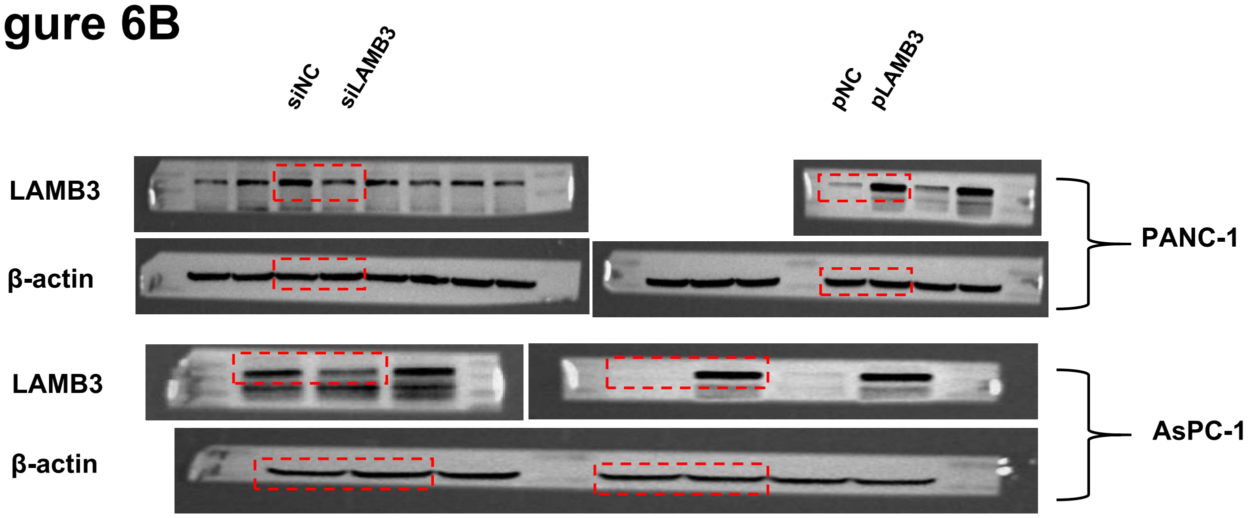

Figure 7A

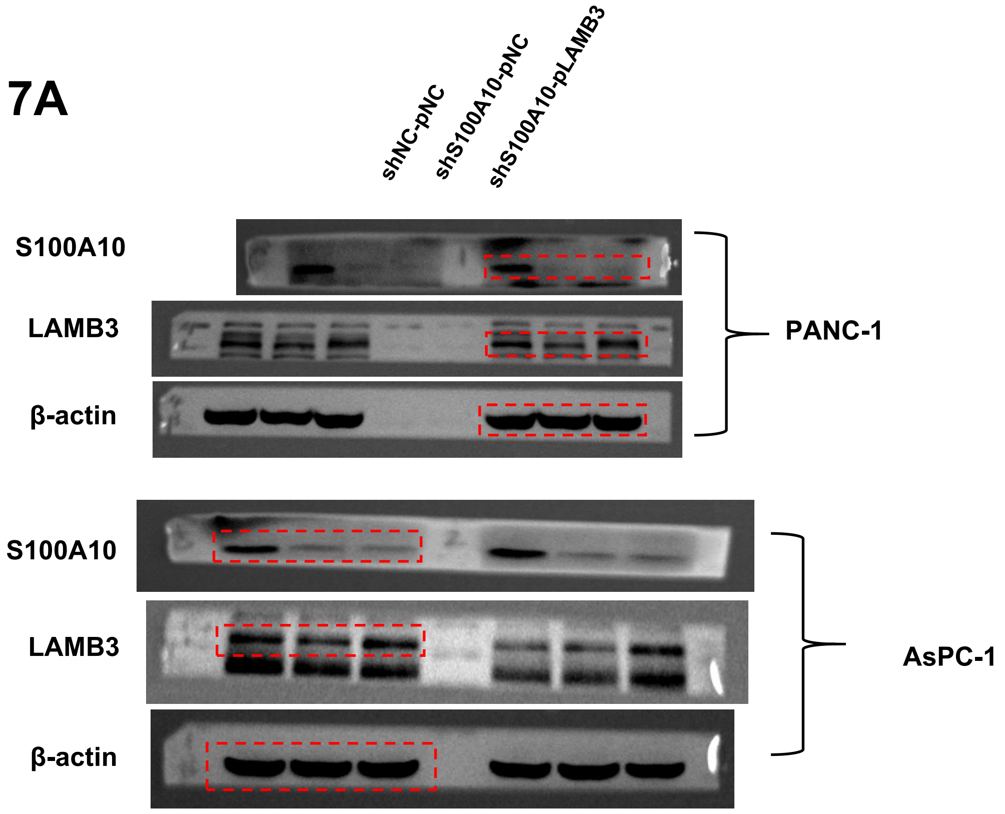

Figure 8A

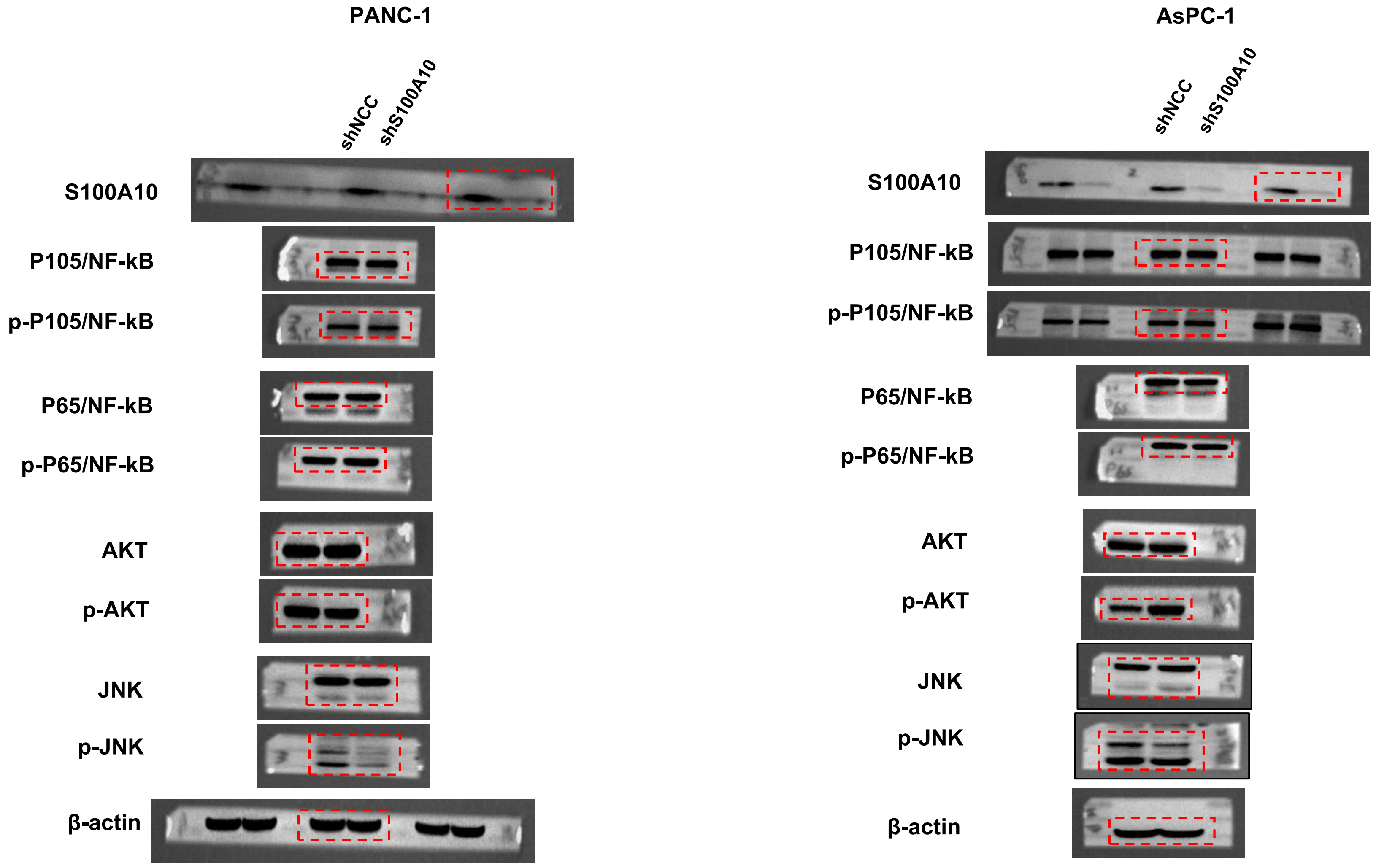

Figure 8B

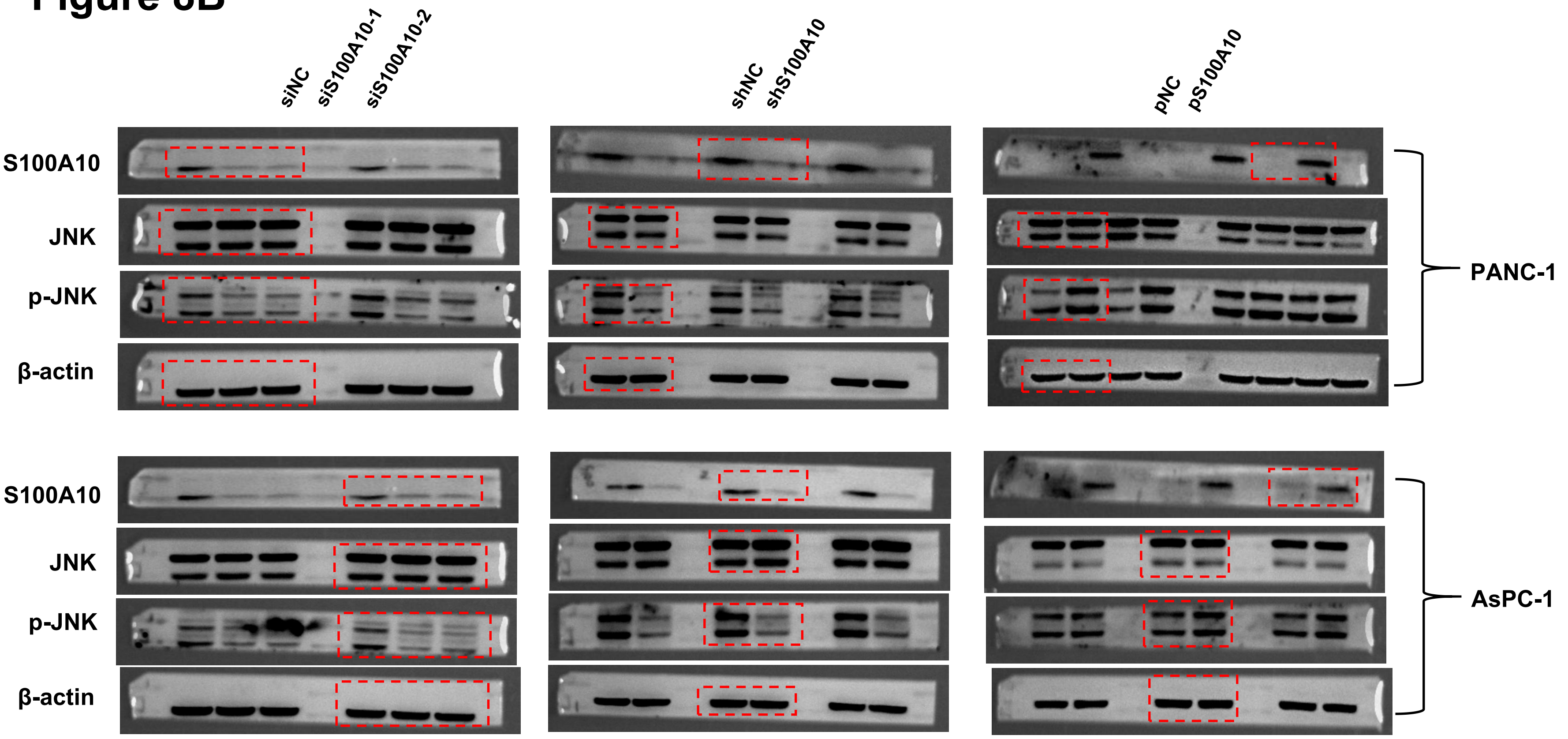

Figure 8C

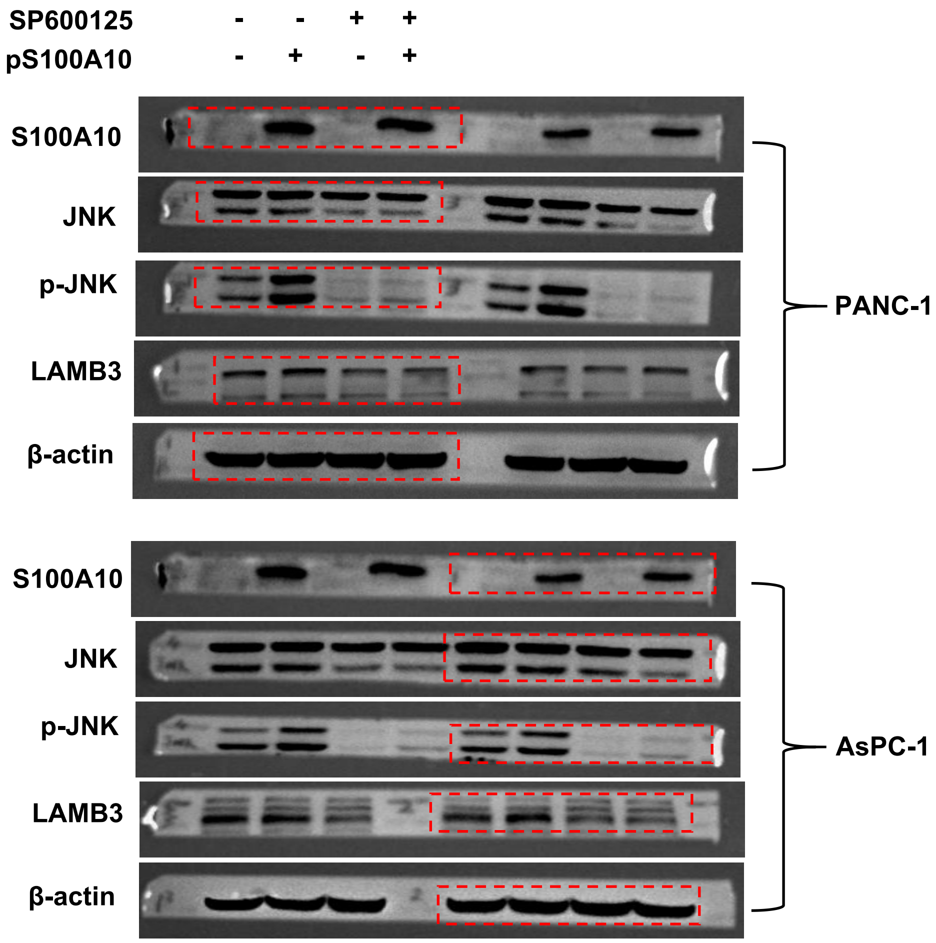

Figure 8D

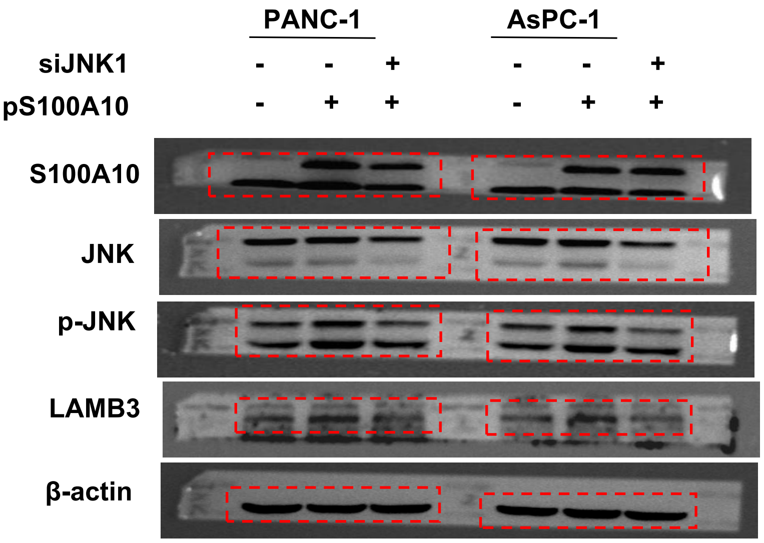

Figure 8H

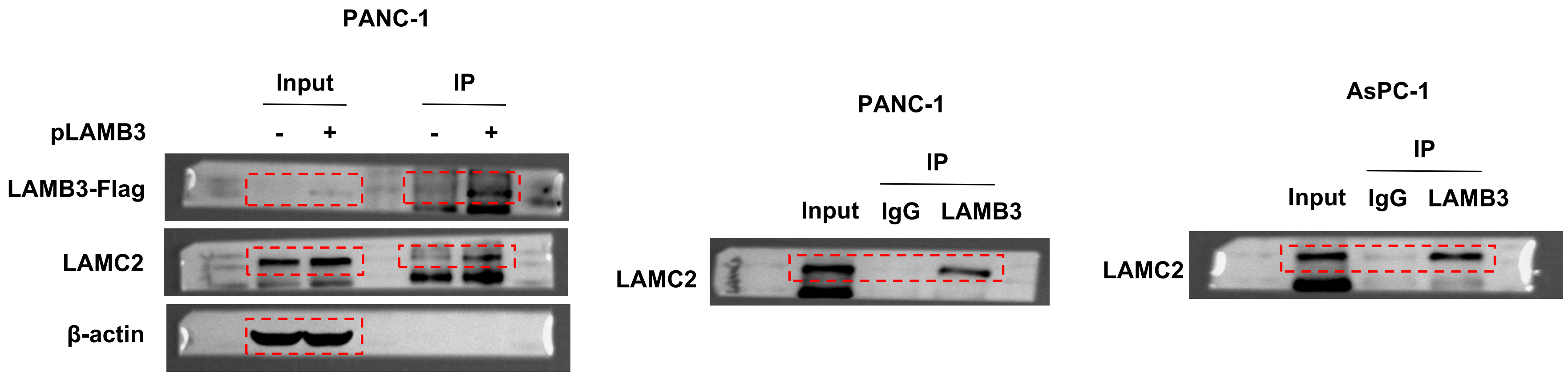

Figure S4H

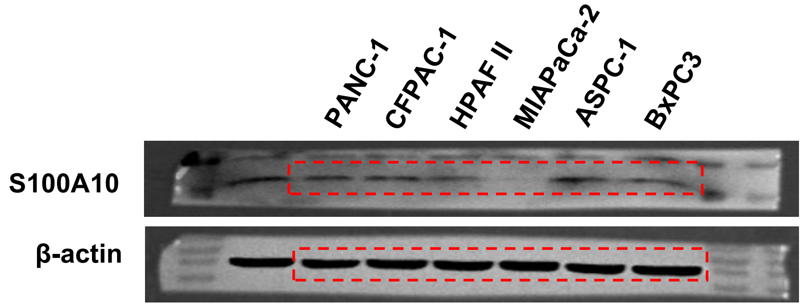

Figure S6A-C

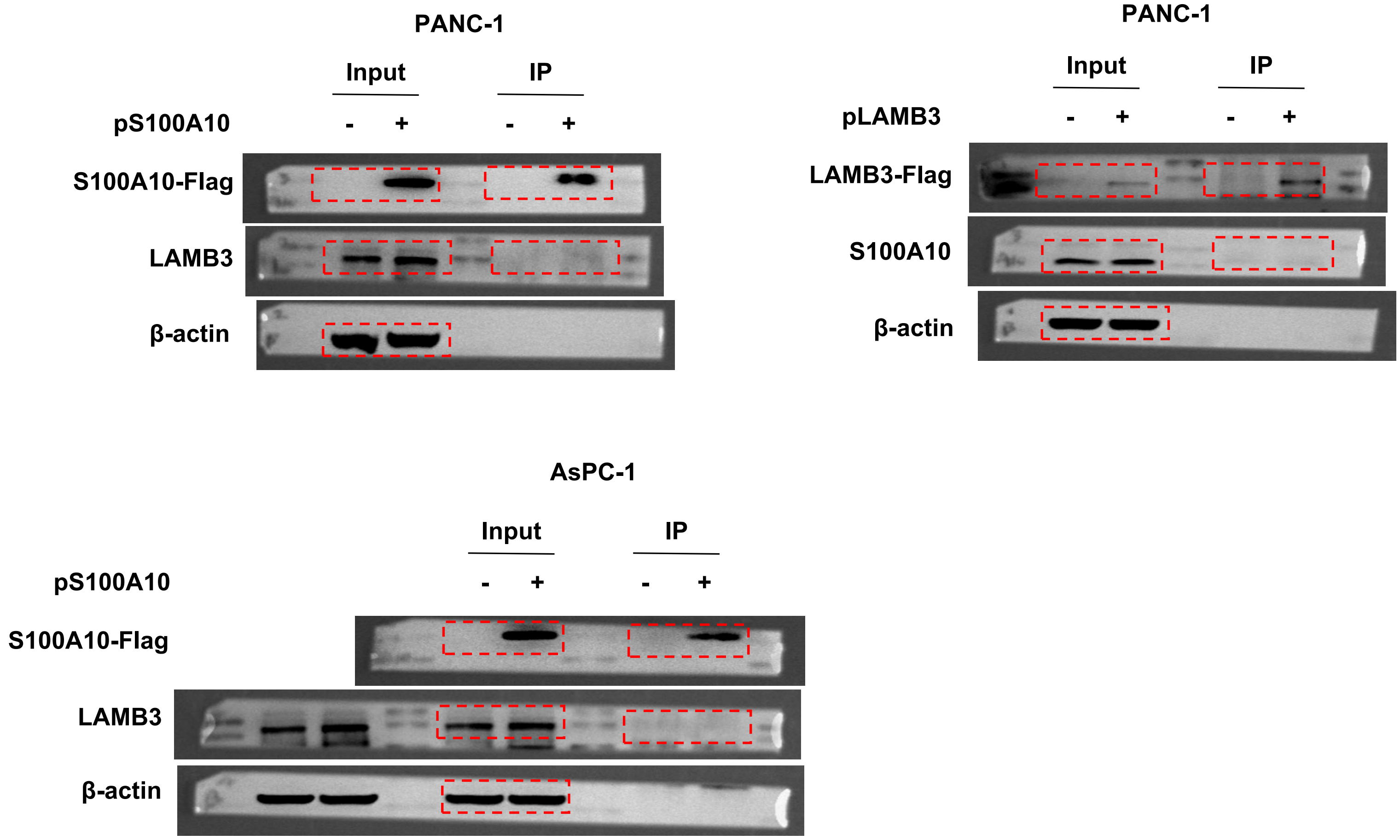

Figure S7. Whole western blot images.
