# Supplementary material for: S100A10 Promotes Pancreatic Ductal Adenocarcinoma Cells Proliferation, Migration and Adhesion through JNK/LAMB3-LAMC2 Axis
Source: Cancers (Basel). 2022 Dec 29;15(1):202. doi: 10.3390/cancers15010202 (PMC9818352; doi:10.3390/cancers15010202)
Supplement: Supplementary file 1 [file cancers-15-00202-s001.zip › Table S1-5 (Proofread).pdf]

Table S1. Oligonucleotide sequences for siRNA

Oligonucleotides	Sequences (5’-3’)
siS100A10-1	GGATAAAGGCTACTTAACA
siS100A10-2	CCATGATGTTTACATTTCA
siLAMB3	GGTGTCAGCTGCACTATTT
siJNK1	GGTAATAGATGCATCTAAA

Table S2. The clinicopathological characteristics of 43 PDAC patients

Characteristic	No. of patients	Percentage
**Total**	**43**	**100%**
**Age**		
Mean ± SD	60.56±11.03	
Median (range)	68 (56-85)	
**Sex**		
Male	26	60.5%
Female	17	39.5%
**Primary site**		
Pancreatic Head	29	67.4%
Pancreatic Body-Tail	14	32.6%
**Grade**		
Moderately differentiated	19	44.2%
Moderately-Poorly differentiated	14	32.6%
Poorly differentiated	10	23.2%
**AJCC TNM stage**		
I /II	12	27.9%
III/IV	9	20.9%
Unknown	22	51.2%
**Radical surgery**		
Yes	37	86.0%
No	6	14.0%

PDAC: pancreatic ductal adenocarcinoma; **SD:** standard deviation.

Table S3. Primers for RT-qPCR

Gene name	Primer	sequence
S100A10	Forward	AAAATCAAAAAGACCCTCTGGC
	Reverse	TAGGGAAAAGAAGCTCTGGAAG
LAMB3	Forward	GAGCCTGTGACTGTGATTTCC
	Reverse	GGTAGCGATTACAGTAGCCTC
ACTB	Forward	CCTGGCACCCAGCACAAT
	Reverse	GGGCCGGACTCGTCATAC
GAPDH	Forward	ACAACTTTGGTATCGTGGAAGG
	Reverse	GCCATCACGCCACAGTTTC

Table S4. Survival analyses for 55 genes in PDAC in GEPIA database

Gene symbol	Overall Survival		
	HR	logrank *p*-value	Group cutoff
ADAM9	2.2	0.00031	Median
LAMA3	2.1	0.00031	Median
COL17A1	2.1	0.00039	Median
INPP4B	2.0	0.00081	Median
ITGA2	2.0	0.0015	Median
LAMB3	1.9	0.0022	Median
IL1RAP	1.9	0.0027	Median
DHRS9	1.9	0.0028	Median
KRT19	1.8	0.0039	Median
SLC2A1	1.8	0.0043	Median
ECT2	1.8	0.005	Median
AHNAK2	1.8	0.0056	Median
ITGA3	1.8	0.0072	Median
SERPINB5	1.8	0.0074	Median
PADI1	1.7	0.008	Median
CAPG	1.7	0.0085	Median
TMC7	1.7	0.0088	Median
ANLN	1.7	0.013	Median
ANO1	1.7	0.013	Median
IFI27	1.7	0.013	Median
S100A14	1.7	0.014	Median

TMPRSS4	1.7	0.014	Median
DCBLD2	1.7	0.015	Median
DSG3	1.7	0.015	Median
KRT6A	1.7	0.015	Median
APOL1	1.6	0.017	Median
AREG	1.6	0.019	Median
NT5E	1.6	0.023	Median
CDH3	1.6	0.027	Median
SLC7A2	0.63	0.027	Median
MMP1	1.6	0.028	Median
IGFL2	1.6	0.029	Median
FERMT1	1.6	0.03	Median
EPHX2	0.64	0.031	Median
MMP14	1.6	0.033	Median
KRT17	1.5	0.036	Median
MYOF	1.5	0.037	Median
PLAU	1.5	0.048	Median
HK2	1.5	0.053	Median
OSBPL3	1.5	0.057	Median
TRIM29	1.5	0.06	Median
SCEL	1.5	0.064	Median
PLEK2	1.5	0.068	Median
ITGB4	1.4	0.078	Median
ADGRF1	1.4	0.09	Median
SERPINB3	1.4	0.1	Median
TNS4	1.3	0.15	Median
LAMC2	1.3	0.19	Median
SLPI	1.3	0.19	Median
CEMIP	1.3	0.21	Median
CEACAM6	1.3	0.22	Median
TTN	0.82	0.37	Median
EGLN3	1.2	0.4	Median
MBOAT2	1.1	0.5	Median
BTG2	1.0	0.83	Median

PDAC: pancreatic ductal adenocarcinoma**; HR:** hazard ratio.

Table S5. Antibodies information for immunohistochemistry, western blotting, Co-Immunoprecipitation (Co-IP) and immunofluorescence

Antibodies	SOURCE	Cat No
S100A10	Proteintech	11250-1-AP
LAMB3	Abcam	ab97765, ab150385
ITGA2	Abcam	ab181548
β-actin	Cell Signaling Technology	3700s
Flag	Cell Signaling Technology	14793S, 8146S
p105/NF-kB	GeneTex	GTX110585-S
p-p105/NF-kB	Cell Signaling Technology	4806S
p65/NF-kB	Cell Signaling Technology	8242
p-p65/NF-kB	Cell Signaling Technology	3033T
AKT	Cell Signaling Technology	4691T
p-AKT	Cell Signaling Technology	4060T
SAPK/JNK	Cell Signaling Technology	9252T
p-SAPK/JNK	Cell Signaling Technology	4668T
LAMC2	Abcam	ab210959
Alexa Fluor®488	ThermoFisher	A-11008
Alexa Fluor™594	ThermoFisher	A-11005
